# Juvenile myoclonic epilepsy heterogeneity uncovered: Z-mapped imaging endophenotypes of cortical and subcortical structures and their clinical, cognitive and psychiatric features

**DOI:** 10.1093/braincomms/fcag107

**Published:** 2026-03-24

**Authors:** Aaron F Struck, Camille Garcia-Ramos, Vivek Prabhakaran, Veena Nair, Anusha Adluru, Santiago Philibert-Rosas, Dace N Almane, Nagesh Adluru, Jana E Jones, Bruce P Hermann

**Affiliations:** Department of Neurology, Washington University in St. Louis School of Medicine, St. Louis, MO 63110, USA; Department of Neurology, University of Wisconsin School of Medicine and Public Health, Madison, WI 53792, USA; Department of Neurology, William S Middleton Veterans Administration Hospital, Madison, WI 53705, USA; Department of Neurology, University of Wisconsin School of Medicine and Public Health, Madison, WI 53792, USA; Department of Radiology, University of Wisconsin School of Medicine and Public Health, Madison, WI 53792, USA; Department of Radiology, University of Wisconsin School of Medicine and Public Health, Madison, WI 53792, USA; Department of Radiology, University of Wisconsin School of Medicine and Public Health, Madison, WI 53792, USA; Department of Neurology, Washington University in St. Louis School of Medicine, St. Louis, MO 63110, USA; Department of Neurology, University of Wisconsin School of Medicine and Public Health, Madison, WI 53792, USA; Department of Neurology, University of Wisconsin School of Medicine and Public Health, Madison, WI 53792, USA; Department of Radiology, University of Wisconsin School of Medicine and Public Health, Madison, WI 53792, USA; Waisman Center, University of Wisconsin-Madison, Madison, WI 53705, USA; Department of Neurology, University of Wisconsin School of Medicine and Public Health, Madison, WI 53792, USA; Department of Neurology, University of Wisconsin School of Medicine and Public Health, Madison, WI 53792, USA

**Keywords:** juvenile myoclonic epilepsy, imaging endophenotypes, neurodevelopment, motor thalamus

## Abstract

To identify imaging endophenotypes in juvenile myoclonic epilepsy using individualized Z-maps of cortical/subcortical regions and examine their relationships with cognitive, psychiatric and epilepsy-related variables. Sixty-two juvenile myoclonic epilepsy patients (aged 12–25 years) and 41 age- and sex-matched healthy controls underwent 3T MRI, neuropsychological assessment, psychiatric evaluation and clinical interviews. Cortical thickness and subcortical volumes were processed with FreeSurfer, adjusted for age, sex and brain volume. Kolmogorov–Smirnov tests were used to compare regional distributions. Z-scores were calculated relative to healthy controls, and K-means clustering identified endophenotypes. In juvenile myoclonic epilepsy, there were reduced subcortical volumes most prominently in motor-related thalamus (*P* < 0.001) and greater variability in cortical thickness in the frontal/parietal regions. Three endophenotypes emerged: subcortical reduction (*n* = 27, 43.5%), increased cortical thickness (*n* = 21, 33.9%) and decreased cortical thickness (*n* = 14, 22.6%). Subcortical reduction showed marked motor thalamic and subcortical grey matter loss. Increased cortical thickness exhibited frontal and parietal cortical thickening with associated subcortical reduction. Decreased cortical thickness showed less subcortical change, but overall reduced cortical thickness. Cognitive differences were notable: Increased Cortical Thickness was the most impaired cognitively, suggesting a disruption of neurodevelopment, while decreased cortical thickness performed the best, particularly in speed/response inhibition—consistent with the least disruption of brain maturation and dysregulation of synaptic pruning. Seizure burden, socioeconomic status, age of onset and psychiatric diagnoses did not show any differences between groups. Juvenile myoclonic epilepsy exhibits heterogeneous imaging endophenotypes, with motor thalamic and subcortical reductions and variability cortical thickness in the frontal and parietal regions, reflecting neurodevelopmental dysregulation with cognitive consequences.

## Teaser

Struck *et al*. applied individualized Z-maps to MRI in 62 juvenile myoclonic epilepsy patients versus 41 controls, revealing motor thalamic/subcortical reductions and three imaging endophenotypes. The increased cortical thickness subgroup showed the poorest cognition, suggesting neurodevelopmental dysregulation, whereas decreased cortical thickness performed best; seizure burden and psychiatric diagnoses did not differ.

## Graphical Abstract

**Graphical Abstract:**
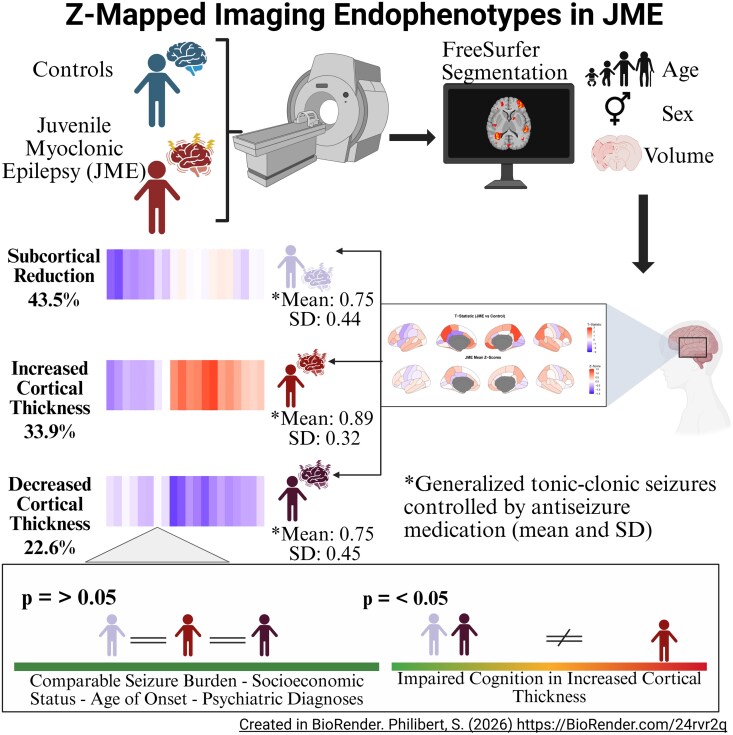

## Introduction

Juvenile myoclonic epilepsy (JME) is the most common adult idiopathic generalized epilepsy,^1-3^ typically lifelong^4^ with 20–30% refractory to medical management.^5^ JME patients face poorer outcomes and increased cognitive/psychiatric comorbidities relative to healthy individuals.^6,7^ Its pathophysiology is elusive; understanding aetiology, neurodevelopment and clinical course may improve therapeutic interventions.

Early genetic research into JME initially identified single-gene mutations in EFHC1 gene, which heighten neuronal excitability.^8^ However, such single-gene mutations are rare, leaving most JME instances without a clear monogenic cause.^9^ Advancements in genetic research have since shifted focus to a polygenic model, leveraging genome-wide association studies to uncover numerous common genetic variants linked to JME.^10,11^ Janz, in his original theories on the pathogenesis of JME, suggested a neurodevelopmental cause resulting in cortical microdysgenesis.^12^ There may be some overlap between these hypotheses, with a broad, mostly polygenic basis that leads to heightened risk of cortical dysgenesis, which in turn leads to hyper-synchronization and hyper-excitability of the epileptic network underlying JME seizure generation. In fact, EFHCmm1 is not only involved in calcium homeostasis, but its overexpression can induce neuronal apoptosis.^8^

Another unexplained clinical finding in idiopathic generalized epilepsies (IGE), of which JME is an example, is its development during adolescence. Often, JME presents as the first type of epilepsy, but can be preceded by childhood absence epilepsy.^13^ We have proposed a neurodevelopmental hypothesis^6,14^ suggesting that dysregulated synaptic pruning in adolescence explains the manifestation of seizures in this age group and also explains the presence of seizures within hours of awakening, as slow-wave sleep is also a time of synaptic pruning and rebalancing of neuronal connections.^15^ The loss of pyramidal inputs to interneurons in the peri-motor cortex, resulting in hyper-synchronization mediated by these interneurons, may be a common pathway leading to myoclonic and generalized tonic-clonic seizures development in adolescence.

Neuroimaging of cortical and subcortical regions has consistently revealed decreased subcortical grey matter volumes, particularly in the thalamus,^16,17^ but investigation of subcortical structures has been limited relative to studies focused on cortical findings. However, findings in cortical regions have been mixed, with cohort studies reporting both increases and decreases in cortical thickness across various areas.^18-20^ We hypothesize that these findings are related to individual heterogeneity, genetic risk and neurodevelopment, which lead to individual differences in cortical thickness that undergo further dysgenic development during adolescence, with the pressure of synaptic pruning. We have shown this relationship within the premotor ventral thalamic network and its relationship to a motor proficiency task.^21^ In this study, we aim to define patient-specific patterns of cortical and subcortical dysgenesis using z-score maps obtained from age- and sex-matched controls to (i) determine the degree of heterogeneity within cortical and subcortical regions of patients with JME, (ii) use unsupervised machine learning to determine if any endophenotypes emerge, (iii) examine the relationship of identified endophenotypes to seizure burden, seizure control and age of onset and (iv) assess their relationship to cognitive and psychiatric comorbidities.

## Materials and methods

### Participants

The participants are from the JMECP (Juvenile Myoclonic Epilepsy Connectome Project). Inclusion criteria for JME (*N* = 63) included a diagnosis supported by at least two of the three following criteria: (i) clinical description or directly observed early morning myoclonic jerks, (ii) clinical description or directly observed generalized tonic-clonic seizures, (iii) an EEG with bursts of 3.5–5 Hz generalized spike-wave and/or polyspike wave discharges, as well as all of the following: (iv) age between 12 and 25 years, (v) English speaking, and (vi) Verbal and Performance IQ ≥ 80. Exclusion criteria included (i) inability to provide informed consent, (ii) reported or directly observed semiological or EEG features that suggest focal epilepsy, (iii) presence of any lesions other than non-specific white matter abnormalities on 3 Tesla MRI with a dedicated epilepsy protocol that includes high-resolution axial and coronal FLAIR sequences, and (iv) an active infectious aetiology of seizures. The control participants were healthy, age and sex-matched to JME participants from other ongoing epilepsy projects or community-based recruitment (via posters and email blasts). Clinical factors (e.g. seizure frequency, age of onset), sociodemographic characteristics (e.g. age, sex, education) were obtained from a structured clinical interview with the patient or the patient’s guardian/parent. All study procedures were reviewed and approved by the University of Wisconsin-Madison Institutional Review Board (IRB) of the UW Human Research Protection Program. All participating parent(s)/guardian(s) signed a designated parent consent form for their involvement in the research study. In addition, all participants aged 18 or older provided written informed consent, for minors (aged 12–17), written parental/guardian consent was obtained in accordance with the IRB protocol.

### MRI acquisition and preprocessing

All JME patients underwent a single 60-min 3T MRI scanning session, which included high-resolution T1 and T2 structural MRI. 3T MRI Hardware: Scanning was done on a GE SIGNA™ Premier 3T wide-bore MRI scanner with a 48-channel head coil designed for high SNR brain imaging and maximum patient compatibility. Parameters for *T*_1_-weighted images were as follows: repetition time (TR)/echo time (TE) = 604 ms/2.516 ms, inversion time (TI) = 1060.0 ms, flip angle = 8°, field-of-view (FOV) = 25.6 cm, 1 mm isotropic; parameters of Cube *T*_2_-weighted images were as follows: TR/TE = 2500 ms/94.641 ms, flip angle = 90°, FOV = 25.6 cm, 1 mm isotropic. Healthy controls (HCs) underwent the same MRI acquisition protocol on the same scanner using identical sequences and parameters.

*T*_1_-weighted images were B1 bias corrected using the N4 correction algorithm^22^ implemented in ANTS. These preprocessed images were then processed using the recon-all pipeline (motion correction, non-uniform intensity normalization, Talairach transform computation, skull stripping, automated subcortical segmentation) in FreeSurfer (http://freesurfer.net) (version 7.4.1). Volumes of target subcortical structures (thalamus, putamen, hippocampus, amygdala, caudate, cerebellum, pallidum, ventral diencephalon, nucleus accumbens) were obtained from the FreeSurfer automated segmentation processing stream and parcellation using the Desikan–Killiany (DK) atlas. Thalamic nuclei volumes were obtained using the process outlined in Iglesias *et al.*^23,24^ Thalamic nuclei and subcortical structures were normalized by dividing their volumes by the estimated total intracranial volume.

### Adjusting for age, sex and brain volume

In the analysis of brain region volumes derived from the DK parcellation via the FreeSurfer pipeline, normalization techniques were applied to controls and patients with JME to mitigate the confounding effects of sex, age and total brain volume (BrainSegVol). Using the mgcv package in R, a Generalized Additive Model (GAM) was fitted for each region, incorporating a smoothing term for age (five knots) to model non-linear age-related changes, alongside linear terms for BrainSegVol and sex (as a factor). Corrected volumes were computed by subtracting the predicted covariate effects, enabling precise comparisons between groups by isolating disease-specific structural differences. This approach aligns with recent neuroimaging studies that leverage GAMs to flexibly model complex, non-linear relationships in brain morphometry, enhancing the detection of subtle abnormalities in epilepsy research.^25-27^

### Z-scoring JME patients and HCs

To ensure effective z-scoring of brain region volumes derived from the DK segmentation in controls, JME patients had normality assessed using skewness (threshold of |skew| < 1), excess kurtosis (threshold of |kurtosis| < 3), and Shapiro–Wilk tests (*P* > 0.05), applied to control data to confirm that z-scored distributions approximate normality. Leave-one-out (LOO) z-scoring was used for controls to avoid bias, while JME patients were z-scored against the full control distribution. These techniques ensure that z-scores reflect standardized deviations from the control mean, facilitating the detection of structural differences. Similar approaches, including normality checks and LOO z-scoring, have been employed in recent neuroimaging studies to normalize data and identify subject-specific abnormalities.^28,29^

### Comparison of distributions

Regions from the DK atlas parcellation and FreeSurfer segmentation regions were combined into lobar-level regions with means across all incorporated regions for thickness measurements and the sum of volumes for segmentation regions (Supplementary Section 1). Probability distribution plots were generated using the ‘geom_density’ function in ggplot, and the distributions were compared statistically using the Kolmogorov–Smirnov (K–S) test, presented both with uncorrected and false discovery rate^30^ corrected *P*-values.

### Unsupervised machine learning

Hierarchical and K-means Clustering were performed using the z-scored measures of the combined regions, as in Supplementary Section 1, for the JME participants. Three methods were used to determine the optimal number of clusters: ‘elbow method’, ‘Gap Statistic’ and bootstrapped (500 trials) Jaccard coefficient^31^ to ensure cluster stability. Clusters from 2 to 5 were investigated.

### Comparison between groups

Imaging endophenotypes were determined, and the resulting groups were then statistically compared for demographic, socioeconomic, cognitive, psychiatric and epilepsy related variables.

### Code availability

Custom analysis scripts are available from the corresponding author upon reasonable request, subject to institutional and data-governance approvals.

## Results

### Participant characteristics

A total of 63 patients with JME and 41 healthy unrelated controls were compared, with no significant differences in age or sex. One JME subject was removed as an outlier, as the total thalamic volume was more than three standard deviations above the mean for the combined JME and control cohorts. This participant’s thalamic volume was 4.2 SD above the mean, while no other participant had an absolute value >2.8 SD from the mean, suggesting either a statistical outlier or an imaging/processing failure that would skew group-level comparisons. After excluding the outlier, a total of 62 JME subjects were used in analyses. Table 1 presents demographic and background information on participants.

**Table 1:** Demographic and background characteristics of JME participants and HCs

	Juvenile Myoclonic epilepsy	Controls	*P*-value
# of participants	62	41	
Age at imaging (years)	20.4 (SD 3.6)	20.5 (SD 3.2)	0.86
Sex (% female)	40 (64.5%)	22 (53.7%)	0.31
Left-handed	4 (6.5%)	1 (2.4%)	0.65
Age of onset (years)	14.1 (SD 4.6)		
Duration (years)	6.3 (SD 5.6)		
Family history epilepsy	3 (4.8%)		
Most recent GTC (months ago)	24.3 (SD 29.0)		
GTC controlled by medications	44/55 (80%)		
# of anti-seizure medications (ASM)	1.48 (SD 0.62)		

### Comparison of regional distributions

**Figure 1:**
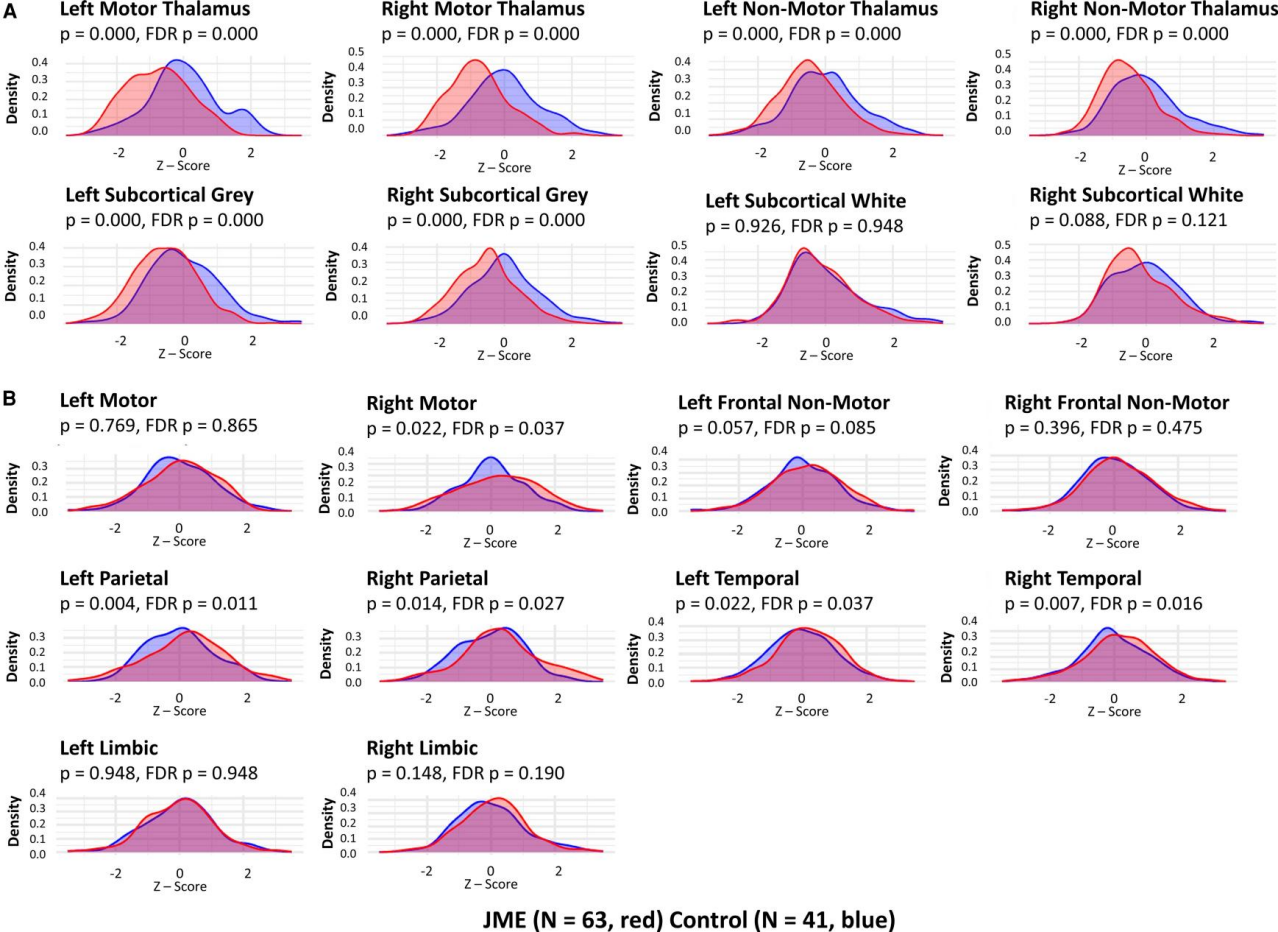
**Regional Z-score distributions in JME and controls.** Overlaying density plots for JME participants regional z-scores (JME *N* = 62, red; controls *N* = 41, blue) after correcting for age, sex and brain volumes. Statistical comparison of distributions is performed with Kolmogorov–Smirnov Test presented with uncorrected and FDR (Benjamini–Hochberg method) correct *P*-values for the **(A)** subcortical and **(B)** cortical regions as defined in Supplementary Section 1.

**Figure 2:**
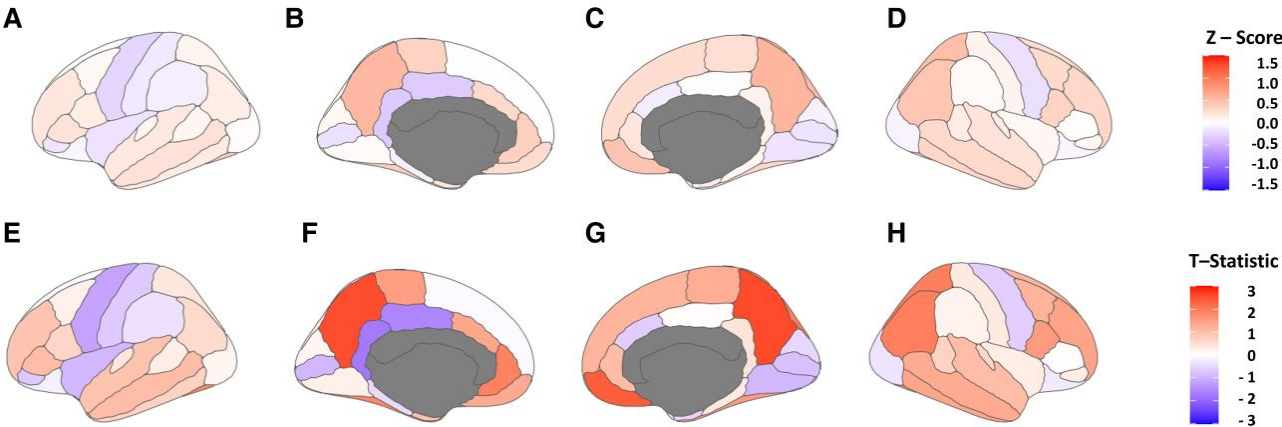
**Cortical mean-Z scores and regional T-statistics (JME versus control).** Surface heatmaps of the mean Z-scored regions for JME (*N* = 62) participants superimposed on Desikan–Killiany (DK) segmentation map as well as the T-statistic comparing those regions to the HCs (*N* = 41). A–D; Mean JME Z-scores: (**A**) left lateral, (**B**) left medial, (**C**) right medial, (**D**) left lateral. E-H; Regional T-Statistics (JME *N* = 63 versus control *N* = 41): (**E**) left lateral, (**F**) left medial, (**G**) right medial, (**H**) right lateral.

After applying non-linear corrections for age, sex and total brain volume, the subcortical volumes and cortical thicknesses were Z-scored relative to HCs. Thalamic nuclei were combined into motor versus non-motor thalamus, and the other subcortical grey and white matter regions were combined. The DK atlas parcellations were averaged across lobes, including parietal, temporal and occipital. However, the limbic system was isolated, and the frontal lobe was divided into pre-motor/motor and the non-motor associated frontal lobes. This was performed for each hemisphere. The distributions were compared between HCs and JME participants with a K–S test with both false discovery rate (FDR)-corrected and uncorrected *P*-values. Figure 1A presents the subcortical regions, and 1B presents the cortical regions. The motor thalamus exhibits a statistically significant bimodal distribution, with the JME patients having a lower volume. The non-motor thalamus and subcortical grey matter volumes were also bimodal and statistically significant, with JME having lower modes. The white matter, occipital, limbic and frontal non-motor regions, as well as the left pre-motor region, showed no statistical differences. The other cortical regions had statistical differences, but instead of a bi-modal distribution, the JME had a wider variance with heavier tails in both directions. These findings suggest, in general, a subcortical reduction in grey matter, maximal in the motor thalamus and a greater heterogeneity in cortical thickness, primarily in the 1-motor associated frontal regions, 2-parietal regions, and 3-temporal regions. Figure 2 is a heatmap superimposed on the cortical surface, showing the mean Z-score for JME participants and the T-statistic when compared with the HC cohort. Supplementary Section 2 shows bar graphs for the mean Z-score and T-statistic for subcortical regions and thalamic nuclei. The overall directionality is towards reduced subcortical volumes, except for the corpus callosum, which was larger (but not significantly) in the JME cohort. Many subcortical regions are significantly reduced, as indicated by FDR-adjusted *P*-values < 0.05 marked with **, with notable bilateral reductions in the hippocampi, cerebellum, accumbens areas and caudate.

### Clustering of Z-score regions

**Figure 3:**
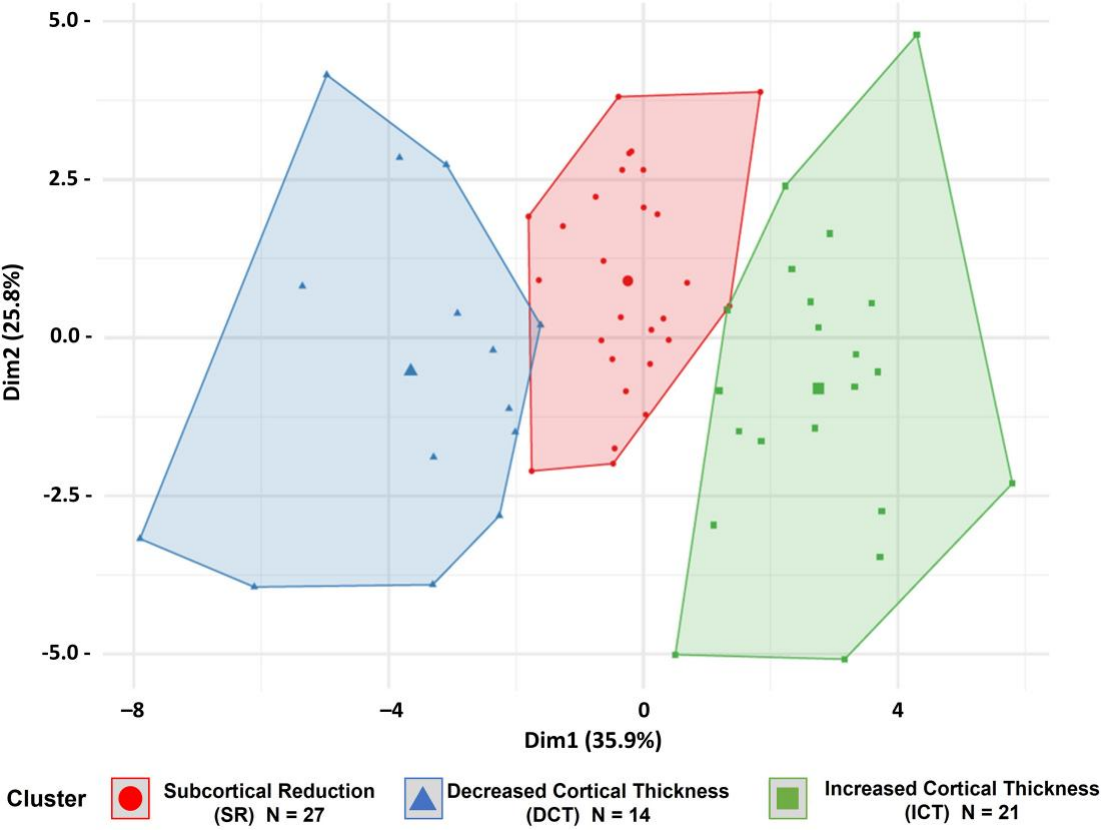
**K-means clustering of JME imaging endophenotypes (K = 3).** Figure shows the separation between K = 3, K-means clusters on the Z-scored regions on the JME patients (each datapoint representing one participant, *N* = 62). This is a 2-D representation based on DIM1: principle component 1 accounting for 35.96% variance and DIM 2: principle component 2 account for 25.8% of the variance. There is minimal overlap between clusters. Cluster sizes were subcortical reduction (SR, *N* = 27); increased cortical thickness (ICT, *N* = 21) and decreased cortical thickness (DCT, *N* = 14).

**Figure 4:**
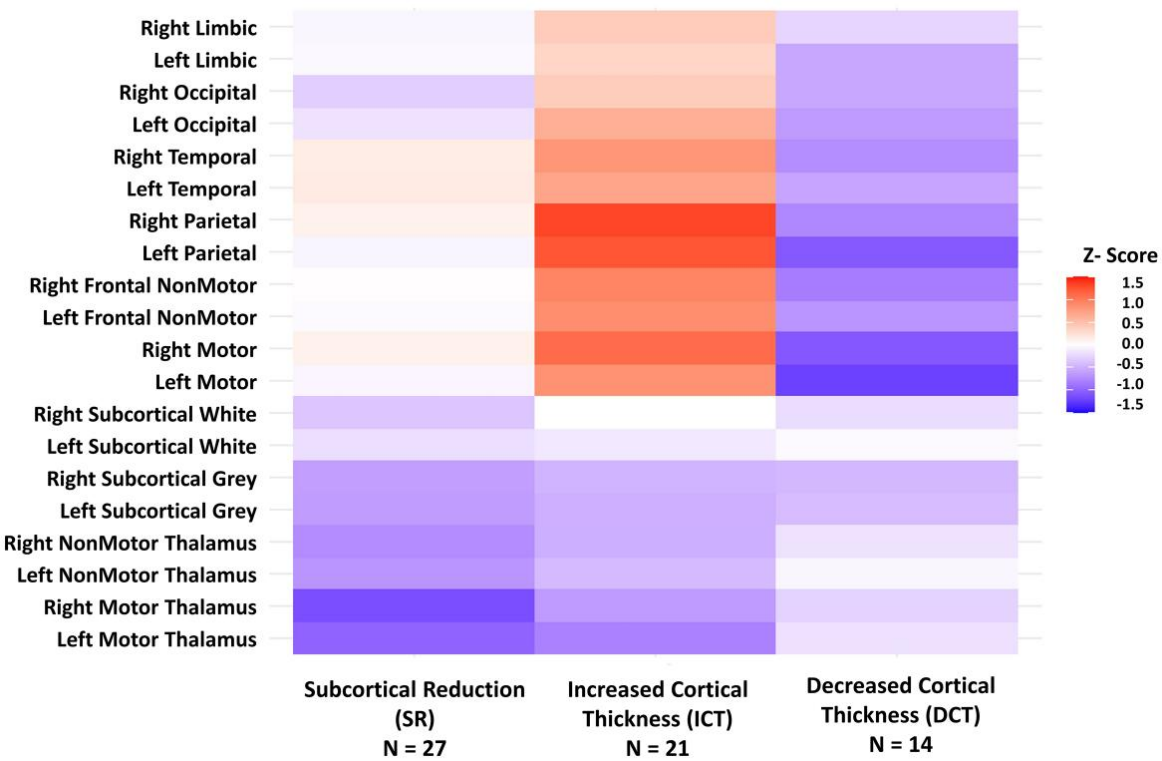
**Cluster-wise mean regional Z-scores across imaging endophenotypes.** Heatmap of mean Z-score for the cortical and subcortical regions for the three imaging groups (endophenotypes): subcortical reduction (SR, *N* = 27), increased cortical thickness (ICT, *N* = 21) and decreased cortical thickness (DCT, *N* = 14). Groups are derived from the Z-scored regions corrected for age, sex and total brain volumes using K-means clustering and the regions are defined in Supplementary Section 1.

Hierarchical and K-means clustering were employed to identify natural variations in patterns of subcortical and cortical changes within JME patients. Clusters between 2 and 5 were assessed. Hierarchical clustering yielded fewer stable clusters, with bootstrapped Jaccard coefficients ranging from 0.49 to 0.56 (Supplementary Section 3). K-means yielded stable clusters at 2 or 3 clusters, with Jaccard coefficients of 0.79 and 0.77, respectively. The Elbow method and Gap Statistic both favoured 3-clusters Supplementary Fig. 1 (A and B) with good separation between clusters, Fig. 3. The clusters were named ‘Subcortical Reduction’ (*n* = 27, 43.5% of total JME group), ‘Decreased Cortical Thickness’ (*n* = 14, 22.6% of total JME) and ‘Increased Cortical Thickness’ (*n* = 21, 33.9% of total JME), based on the relative differences in these findings. The ‘Subcortical Reduction’ had the most significant difference in the right and left motor thalamus and diffuse reduction in subcortical structures, with fewer changes related to cortical thickness. The ‘Increased Cortical Thickness’ group exhibited increased cortical thickness primarily in the right and left parietal and premotor regions, as well as throughout the cortex (Fig. 4). This group also had a reduction in subcortical volumes. The ‘Decreased Cortical Thickness’ group had decreased cortical thickness in similar regions to the ‘Increased Cortical Thickness’ group, suggesting that these regions remain relevant to this group, but the direction of the findings is opposite to the expected direction. This group had the least difference in the subcortical regions. Supplementary Fig. 2 presents regional boxplots and a statistical comparison of three clusters compared with HCs, including uncorrected *P*-values from the Wilcox statistical test.

### Comparison of groups for demographic, cognitive, psychiatric and epilepsy related variables

Table 2 provides a summary of the differences between groups across these variables. The group sizes varied with 27/62 JME patients in the subcortical reduction group, 21/62 in the increased cortical thickness group and 14/62 in the decreased cortical thickness group. Overall, the age ranges were similar with overlapping ranges. There was a marginal difference between the subcortical and increased cortical thickness groups at *P* = 0.049, with the subcortical reduction group being older at the age of 21.39, and the increased cortical thickness group at 19.34 years. The sex distribution was similar between the subcortical and increased cortical thickness groups showing an increased female distribution. In contrast, the decreased cortical thickness group was evenly split between males and females with no statistical difference. The IQ score and Area Deprivation Index^32^ (a measure of socioeconomic status) were not statistically different between the groups; however, a trend (*P* = 0.069) was observed with decreased IQ scores in the increased cortical thickness group and the subcortical reduction group, which became more apparent when using other methods for stratifying cognitive ability.

**Table 2:** JME cluster and pairwise comparisons

Variable	Subcortical reduction	Increased cortical thickness	Decreased cortical thickness	SR versus ICT	SR versus DCT	ICT versus DCT
*N*	*n* = 27	*n* = 21	*n* = 14			
Age (years)	Mean: 21.39 (SD: 3.08), Range: 14.50–25.67	Mean: 19.34 (SD: 3.71), Range: 13.25–24.75	Mean: 20.15 (SD: 4.10), Range: 13.33–25.92	**0**.**0495**	0.3498	0.4287
Gender	Female: 0.70; Male: 0.30	Female: 0.67; Male: 0.33	Female: 0.50; Male: 0.50	1.0000	0.2984	0.4683
Area Deprivation Index	Mean: 50.19 (SD: 19.71), Range: 10.00–79.00	Mean: 46.80 (SD: 24.73), Range: 13.00–96.00	Mean: 46.23 (SD: 14.26), Range: 22.00–75.00	0.5323	0.3703	0.7540
# ASM	Mean: 1.59 (SD: 0.69), Range: 0.00–3.00	Mean: 1.33 (SD: 0.48), Range: 1.00–2.00	Mean: 1.50 (SD: 0.65), Range: 1.00–3.00	0.1431	0.5838	0.4975
GTC controlled by ASMs	Mean: 0.75 (SD: 0.44), Range: 0.00–1.00	Mean: 0.89 (SD: 0.32), Range: 0.00–1.00	Mean: 0.75 (SD: 0.45), Range: 0.00–1.00	0.2384	1.0000	0.3087
Most Recent GTC (months)	Mean: 32.24 (SD: 36.42), Range: 1.00–126.00	Mean: 17.05 (SD: 20.64), Range: 1.00–76.00	Mean: 19.62 (SD: 19.77), Range: 1.00–63.00	0.4911	0.6004	0.6585
Abn EEG despite ASM	Absence: 0.62; Presence: 0.38	Absence: 0.71; Presence: 0.29	Absence: 0.64; Presence: 0.36	0.5537	1.0000	0.6947
Epilepsy duration (years)	Mean: 8.09 (SD: 6.27), Range: 0.50–20.00	Mean: 5.35 (SD: 4.48), Range: 0.50–13.75	Mean: 5.53 (SD: 5.52), Range: 0.83–17.58	0.1454	0.2870	0.9367
Age of onset (years)	Mean: 13.63 (SD: 5.11), Range: 2.67–22.25	Mean: 14.04 (SD: 2.91), Range: 7.50–20.92	Mean: 15.24 (SD: 5.90), Range: 3.67–24.42	0.8111	0.3053	0.3567
Valproic acid use	FALSE: 0.78; TRUE: 0.22	FALSE: 1.00; TRUE: 0.00	FALSE: 0.71; TRUE: 0.29	**0**.**0315**	0.7131	**0**.**0230**
ADHD	False: 0.88; True: 0.12	False: 0.76; True: 0.24	False: 0.79; True: 0.21	0.4428	0.6477	1.0000
Anxiety	False: 0.65; True: 0.35	False: 0.52; True: 0.48	False: 0.79; True: 0.21	0.4028	0.4748	0.1729
Depression	False: 0.38; True: 0.62	False: 0.62; True: 0.38	False: 0.64; True: 0.36	0.1614	0.1979	1.0000
IQ score	Mean: 100.63 (SD: 14.90), Range: 73.00–126.00	Mean: 92.62 (SD: 11.00), Range: 73.00–113.00	Mean: 98.79 (SD: 10.24), Range: 80.00–117.00	0.0687	0.6898	0.1132
General Ability	Mean: −0.01 (SD: 0.95), Range: −2.10–1.63	Mean: −0.64 (SD: 0.75), Range: −1.82–0.52	Mean: −0.09 (SD: 0.69), Range: −1.52–0.87	**0**.**0159**	0.5180	0.0663
Speed/response inhibition	Mean: 0.07 (SD: 0.89), Range: −2.51–1.27	Mean: −0.23 (SD: 1.01), Range: −3.06–1.68	Mean: 0.37 (SD: 0.49), Range: −1.14–1.00	0.2200	0.3427	**0**.**0146**
Lrng/Memory	Mean: −0.14 (SD: 1.00), Range: −2.57–1.73	Mean: −0.35 (SD: 0.90), Range: −2.19–1.08	Mean: −0.31 (SD: 0.55), Range: −1.66–0.28	0.4296	0.5180	0.9597
Cognition: high average	False: 0.67; True: 0.33	False: 0.81; True: 0.19	False: 0.93; True: 0.07	0.3288	0.1314	0.6417
Cognition: averagenormal	False: 0.52; True: 0.48	False: 0.52; True: 0.48	False: 0.07; True: 0.93	1.0000	**0**.**0060**	**0**.**0120**
Cognition: diffusely abnormal	False: 0.81; True: 0.19	False: 0.67; True: 0.33	False: 1.00; True: 0.00	0.3298	0.1404	**0**.**0260**

Bold values indicate statistical significance results (*P* < 0.05).

Cognitive ability was assessed using a previously described^33^ factor analysis on this cohort, which identified three primary cognitive factors: a ‘General Ability’ domain, a ‘Speed/Response Inhibition’ domain and a ‘Learning/Memory’ domain. In the ‘General Ability’ domain, the increased cortical thickness group had the lowest score, and it was statistically significant when compared with the decreased cortical thickness group, which had better ‘Speed/Response Inhibition’ than the other two, reaching statistical significance (*P* = 0.014) when compared with the increased cortical thickness group. The ‘Learning/Memory’ domain was relatively similar between the groups. Cognition was also assessed using previously described cognitive clusters in these groups.^33^ The clusters were loosely, high average (mean IQscore = 112.0), average (mean IQ score= 96.5), and low average/diffusely abnormal in cognition (mean IQ score= 83.2). Compared to the cognitive groups derived from imaging, the increased cortical thickness group had the most participants in the low average/diffusely abnormal group. It showed statistical significance when compared to the other two groups. This group was also more likely to exhibit diffusely abnormal cognition, and this finding was significant when compared to the group with decreased cortical thickness. Lifetime-to-date diagnoses of ADHD, anxiety and depression were not significantly different between any groups [overall rates of ADHD (18% JME, 7% controls, *P* = 0.16), anxiety (36% JME, 34% controls, *P* = 1.0) and depression (JME 48%, controls 27%, *P* = 0.04)].

The epilepsy related variables including number of anti-seizure medications (# ASM), generalized tonic clonic seizure (GTC) suppressed by medications (GTCMEDS), time since most recent GTC, an abnormal EEG despite anti-seizure medication therapy, the duration of epilepsy and the age of onset did not have any differences, except for the use of valproic acid which was significantly less likely in the increased cortical thickness group (0% use) compared to the subcortical reduction group (22% use, *P* = 0.03) and the decreased cortical thickness group (29% use, *P* = 0.19).

In summary, the analysis demonstrates that the imaging-related endophenotypes are largely defined by a reduction in subcortical grey matter and by differences in cortical thickness (increased or decreased), primarily in motor-related frontal, parietal and temporal regions, with no clear differences in epilepsy-related variables. Meaningful differences are observed in cognition but not psychiatric status. In general, the increased cortical thickness group exhibited the most significant cognitive deficits primarily centred around general cognitive ability. The decreased cortical thickness group performed the best cognitively, particularly in the domain of speed and response inhibition.

## Discussion

The goal of this study was to characterize the presence and nature of individual-level heterogeneity within JME that might explain the conflicting reports of both increased and decreased thickness across the cortex.^18,19^ We hypothesize that JME is a network disease characterized by a typical phenotype of clinical signs/symptoms (onset in adolescence, early morning myoclonus, generalized epileptiform discharges), but that the underlying neurodevelopmental pathophysiology that leads to this clinical presentation can vary, as suggested by heterogeneity in cognitive profiles.^19,33^ The emergence of generalized polyspike-waves and seizures during adolescence also suggests a role of synaptic pruning of the motor-related cortico-thalamic networks as key to the JME phenotype. In previous work, we have demonstrated that improved motor performance in JME and HCs is associated with decreased cortical thickness of the motor-related cortex and the ventral (motoric) thalamus.^21^ We hypothesize that there may be distinct patterns of neurodevelopmental dysregulation within JME with core involvement of the motor thalamus and motor-related cortex that result in the classical clinical phenotype, but variability beyond that core network, resulting in differences in cognitive profiles and asymmetry of seizure onsets.^34,35^

To test this hypothesis, after adjusting for age, sex and brain volume, we Z-scored cortical and subcortical regions relative to the HCs. We first looked at the distributions of the Z-scores of the JME group and found that (i) JME patients had a reduction in subcortical grey matter, but not white matter volume. This reduction was most evident in the motor-related nuclei of the thalamus, but was quite diffuse, including the cerebellum. (ii) In the cortical regions, there was a different finding. What was more evident than a clear directionality in cortical thickness was a difference in the variance of the distributions. The frontal-motor and parietal regions were the most likely to diverge from the HCs. However, the affected cortical regions could be broader, sometimes extending into non-motor frontal and temporal regions. Heterogeneity can be found even in the laterality of these cortical findings.^14^ The distributions, had longer and thicker tails in both directions suggesting higher kurtosis, which implies that JME patients were more likely to have both increased and decreased cortical thickness, consistent with deregulation of neurodevelopment. These results suggest a core network characterized by relatively symmetric involvement of the motor thalamic nuclei, accompanied by more asymmetric yet essential involvement of the peri-motor cortex, and variable engagement of broader cortical regions. This model illustrates a shared network that results in the clinical phenotype of adolescence-onset myoclonic seizures, and as will be seen, can also explain the variance in cognitive profiles and variable neuroimaging findings reported in the literature.

To understand the patterns of this dysregulation, we used unsupervised machine learning. We identified three distinct clusters: one showing a primarily ‘Subcortical Reduction’, one with ‘Increased Cortical Thickness’ mainly in the peri-motor regions accompanied by some degree of decreased volumes in the subcortical regions, primarily in the motor-thalamus. The final group displayed less change within the subcortical regions but had reduced cortical thickness again in the motor-associated frontal cortex and parietal regions. In terms of disease and cognitive-related variables, the cognitive differences were more evident than seizure-related variables, as would be suggested by the neurodevelopmental model of JME, where the disease-related variables are primarily related to the per-motor cortex/subcortical network and the cognitive variables are related to the varying extent of cortical abnormalities that extend beyond this core seizure network.

### Cortical imaging heterogeneity and comparison with cognition and disease-related variables

Structural imaging studies of cortical thickness in JME have yielded inconsistent findings, reflecting the condition’s heterogeneity.^18,19,36,37^ Some studies report increased cortical thickness in medial frontal and pre-motor regions, as detected via voxel-based morphometry^18,36^ while others identify decreased thickness in frontal areas, often associated with cognitive impairments.^19,37^ These discrepancies may arise from individual variability in neurodevelopmental processes, medication effects or age-related changes.^6,38^ To highlight non-age-related differences in the neuroimaging, we used a non-linear correction to generate individualized Z-maps. From this, we identified three distinct imaging endophenotypes with the following frequencies: subcortical reduction (43.5%), increased cortical thickness (33.9%) and decreased cortical thickness (22.5%)—highlighting the variability in cortical thickness, particularly in motor-related frontal, parietal and temporal regions. We propose that these differences stem from a fundamental neurodevelopmental anomaly in the motor-associated cortical regions, but that the neurodevelopmental dysregulation may and in fact often does extend beyond those regions. Genetic studies have not identified consistent monogenic causes for JME, though rare familial cases implicate EFHC1, a microtubule-associated protein involved in neuronal migration.^8,9,39^ Other candidate genes, such as those encoding ion channels, may contribute to dysgenesis in specific cortical regions, as observed in other epilepsies, including nicotinic acetylcholine receptor epilepsy (frontal lobe) and LGI1-mediated epilepsy (temporal lobe).^40-42^ The observed cortical thickness heterogeneity in our endophenotypes, particularly in motor/parietal regions, likely reflects individual differences in neurodevelopmental dysregulation modulated by polygenic risk and age-related pruning, while involvement of the motor and thalamic networks ensures a consistent clinical phenotype across patients.

In idiopathic generalized epilepsies such as JME, postmortem studies have described subtle histological abnormalities, most notably microdysgenesis, supporting a neurodevelopmental substrate for these disorders,^12^ yet direct correlations with imaging-detected grey matter changes, including our Z-mapped cortical thickness endophenotypes, remain limited due to ethical barriers in obtaining tissue from young cohorts. Indirect evidence from type 1a focal cortical dysplasia supports potential parallels, with advanced imaging correlating well to pathological confirmation. FreeSurfer-based cortical thickness measures align robustly with histologic validations in epilepsy,^23,24^ while abnormal synaptic pruning has been proposed as a plausible neurodevelopmental mechanism is JME, consistent with the broader literature showing that disruptions in pruning can produce pathological circuitry alterations.^43^

Cognitively, dysexecutive function is the most common^44,45^ but not invariably^46^ noted abnormality in JME. As with neuroimaging findings, mixed reports of cognitive abnormalities may be linked to JME endophenotypes under investigation. Longitudinal studies are needed to disentangle these effects. The decreased cortical thickness group appears to be the least cognitively affected, and the increased cortical thickness group the most adversely affected, which we assume reflects more developmentally appropriate pruning rather than a pathologic loss of tissue.

There were no differences between endophenotypes in terms of age of onset, number of ASMs, abnormal EEG despite ASMs, or time since the most recent GTC. There was a significantly decreased use of valproic acid in the increased cortical thickness group. The meaning of this finding is unclear. Prior studies have demonstrated that valproic acid use is associated with decreased cortical thickness.^38^ Potentially, this is not indicative of direct drug effects but may be related to valproic acid’s efficacy in spike suppression (that could improve cognition) or modifying synaptic plasticity to make it less dysregulated. Valproic acid may not necessarily lead to cortical pathology as the groups with increased valproic acid use have better cognitive outcomes, but the role of anti-seizure medication particularly valproic acid in neurodevelopment remains an understudied and important topic of investigation. Longitudinal studies with larger cohorts and as well as animal models of IGEs may help to understand this question. Further investigation into family members s who have spikes but do not require medications can help disentangle genetic and medication effects from developmental processes.

### Age, sex, psychiatric and socioeconomic factors

The increased cortical thickness group was the youngest, at 19.34 years, and was statistically lower than the subcortical reduction group, but not the decreased cortical thickness group. The age ranges had a significant overlap, so it is unlikely that the imaging differences are solely related to an age effect or inadequate correction for age: there is a complex relationship within these variables that may, in part, be related to disease duration^14,18,47,48^ and patient-specific dysregulation of cortical development and adolescent synaptic pruning. The study’s gender breakdown favoured female participants, which is expected, as JME has a higher prevalence in females. The decreased cortical thickness group had the most balanced sex ratio, but was also the smallest group. The role of gender-specific hormones and neurodevelopmental trajectories in JME needs further study to understand if this result is meaningful. Similarly, there was no difference in the prevalence of current psychiatric diagnoses between the imaging groups. The increased cortical thickness group did have the highest prevalence of deficits in executive function (speed/response inhibition). Within this group, this topic deserves further study, as ADHD is reported at higher rates in JME than the general population^49^ and this is true in the JMECP cohort as well, but was not significant.

### Subcortical differences

Subcortical imaging analyses in our study revealed consistent reductions in grey matter volumes across JME patients, particularly in the thalamus, with the most pronounced effects in the motor-associated nuclei, such as the ventral anterior and ventrolateral nuclei, as well as affecting multiple other structures. The motor thalamus showed statistically significant bimodal distributions, with JME patients having lower volumes compared to controls, as confirmed by K–S tests (Fig. 1A). These findings align with prior studies reporting thalamic volume reductions in JME,^16,17^ but our unsupervised clustering approach highlights heterogeneity in subcortical involvement. The prominent reduction in motor thalamic nuclei suggests a critical role in JME’s pathophysiology, potentially disrupting the cortico-thalamic motor network and contributing to myoclonus. We propose that neurodevelopmental dysregulation, possibly exacerbated by synaptic pruning during adolescence, leads to disinhibition of layer 5/6 pyramidal neurons in the motor-associated frontal cortex. This loss of interneuron-mediated feedback, coupled with impaired thalamic modulation, may facilitate hypersynchronous discharges that propagate via the corpus callosum, resulting in myoclonus and generalized tonic-clonic seizures.^15,50-52^ The Subcortical Reduction group’s pronounced thalamic atrophy underscores a primary subcortical pathology. In contrast, the increased cortical thickness group’s combined subcortical and cortical abnormalities suggest a broader network dysfunction, potentially linked to polygenic risk and neurodevelopmental variability.

### Limitations

This study offers valuable insights into the imaging endophenotypes of JME; however, several limitations must be acknowledged. First, the cross-sectional cohort design limits our ability to capture the dynamic progression of cortical and subcortical changes over time. Longitudinal studies are essential for elucidating how these endophenotypes evolve, particularly in relation to neurodevelopmental processes like synaptic pruning during adolescence, which may drive the observed heterogeneity in cortical thickness and subcortical volumes. Second, while we hypothesize a polygenic basis for JME’s pathophysiology, supported by prior genetic studies,^10,11^ our study did not include genetic profiling of participants. Incorporating genomic data, such as polygenic risk scores or analysis of rare variants like EFHC1, could clarify the relationship between genetic background and imaging endophenotypes. Third, although we applied non-linear corrections for age, sex and brain volume using generalized additive models, these adjustments may be imperfect, especially during adolescence—a period marked by rapid and variable neurodevelopmental changes. The overlapping age ranges across our endophenotypes (subcortical reduction: mean = 21.39 years; increased cortical thickness: mean = 19.34 years; decreased cortical thickness = 20.15 years) suggest potential residual confounding, which could obscure subtle disease-specific effects. Finally, while our findings suggest that motor-associated cortical and thalamic regions are involved in JME’s pathophysiology, non-invasive neuroimaging cannot definitively confirm their functional involvement. Invasive neuromonitoring with intracranial EEG would be necessary to validate the specific roles of peri-motor cortex and motor thalamic nuclei in generating myoclonic jerks and generalized seizures. Future studies addressing these limitations could enhance our understanding of JME’s neurobiological underpinnings and inform the development of targeted therapeutic strategies.

## Conclusion

This study delineates three imaging endophenotypes in JME, characterized by pronounced subcortical reductions in motor thalamic nuclei and heterogeneous cortical thickness alterations in motor-associated frontal and parietal regions, reflecting neurodevelopmental dysregulation and variable cognitive outcomes. The consistent motor thalamus atrophy underscores its pivotal role in the pathophysiology of JME, while cortical thickness variability highlights individual differences in disease expression. Longitudinal studies that integrate genotyping and invasive neuromonitoring are essential for unravelling the dynamic interplay of these structural changes and informing targeted therapeutic strategies.

## Supplementary Material

fcag107_Supplementary_Data

## Data Availability

The data that support the findings of this study are available from the corresponding author upon reasonable request.

## References

[1] Amrutkar CV, Riel-Romero RM. Juvenile myoclonic epilepsy.Statpearls [Internet]. StatPearls Publishing; 2024. [cited 2024 Jul 25]. Available from: http://www.ncbi.nlm.nih.gov/books/NBK537109/

[2] Panayiotopoulos CP, Obeid T, Tahan AR. Juvenile myoclonic epilepsy: A 5-year prospective study. Epilepsia. 1994;35(2):285–296. 8156946 10.1111/j.1528-1157.1994.tb02432.x

[3] Camfield CS, Striano P, Camfield PR. Epidemiology of juvenile myoclonic epilepsy. Epilepsy Behav. 2013;28(Suppl 1):S15–S17. 23756473 10.1016/j.yebeh.2012.06.024

[4] Baykan B, Martínez-Juárez IE, Altindag EA, et al Lifetime prognosis of juvenile myoclonic epilepsy. Epilepsy Behav. 2013;28(Suppl 1):S18–S24. 23756474 10.1016/j.yebeh.2012.06.036

[5] Gelisse P, Genton P, Thomas P, et al Clinical factors of drug resistance in juvenile myoclonic epilepsy. J Neurol Neurosurg Psychiatry. 2001;70(2):240–243. 11160477 10.1136/jnnp.70.2.240 PMC1737198

[6] Lin JJ, Dabbs K, Riley JD, et al Neurodevelopment in new-onset juvenile myoclonic epilepsy over the first 2 years. Ann Neurol. 2014;76(5):660–668. 25087843 10.1002/ana.24240 PMC4362677

[7] Gélisse P, Thomas P, Samuelian J-C, Gentin P. Psychiatric disorders in juvenile myoclonic epilepsy. Epilepsia. 2007;48(5):1032–1033. 17509008 10.1111/j.1528-1167.2007.01009_4.x

[8] Suzuki T, Delgado-Escueta AV, Aguan K, et al Mutations in EFHC1 cause juvenile myoclonic epilepsy. Nat Genet. 2004;36(8):842–849. 15258581 10.1038/ng1393

[9] Annesi F, Gambardella A, Michelucci R, et al Mutational analysis of EFHC1 gene in Italian families with juvenile myoclonic epilepsy. Epilepsia. 2007;48(9):1686–1690. 17634063 10.1111/j.1528-1167.2007.01173.x

[10] Leu C, Stevelink R, Smith AW, et al Polygenic burden in focal and generalized epilepsies. Brain. 2019;142(11):3473–3481. 31608925 10.1093/brain/awz292 PMC6821205

[11] Stevelink R, Campbell C, Chen S, et al GWAS meta-analysis of over 29,000 people with epilepsy identifies 26 risk loci and subtype-specific genetic architecture. Nat Genet. 2023;55(9):1471–1482. 37653029 10.1038/s41588-023-01485-w PMC10484785

[12] Meencke HJ, Janz D. Neuropathological findings in primary generalized epilepsy: A study of eight cases. Epilepsia. 1984;25(1):8–21. 6692795 10.1111/j.1528-1157.1984.tb04149.x

[13] Wirrell EC, Camfield CS, Camfield PR, et al Long-term prognosis of typical childhood absence epilepsy: Remission or progression to juvenile myoclonic epilepsy. Neurology. 1996;47(4):912–918. 8857718 10.1212/wnl.47.4.912

[14] Struck AF, Garcia-Ramos C, Gjini K, et al Juvenile myoclonic epilepsy imaging endophenotypes and relationship with cognition and resting-state EEG. Hum Brain Mapp. 2025;46(7):e70226. 40347042 10.1002/hbm.70226 PMC12063524

[15] Tononi G, Cirelli C. Sleep and the price of plasticity: From synaptic and cellular homeostasis to memory consolidation and integration. Neuron. 2014;81(1):12–34. 24411729 10.1016/j.neuron.2013.12.025 PMC3921176

[16] Pulsipher DT, Seidenberg M, Guidotti L, et al Thalamofrontal circuitry and executive dysfunction in recent onset juvenile myoclonic epilepsy. Epilepsia. 2009;50(5):1210–1219. 19183226 10.1111/j.1528-1167.2008.01952.x PMC2931325

[17] Kim JH, Kim JB, Suh S, Kim DW. Subcortical grey matter changes in juvenile myoclonic epilepsy. Neuroimage Clin. 2018;17:397–404. 29159052 10.1016/j.nicl.2017.11.001 PMC5683808

[18] Alhusaini S, Ronan L, Scanlon C, et al Regional increase of cerebral cortex thickness in juvenile myoclonic epilepsy. Epilepsia. 2013;54(9):e138–e141. 23944956 10.1111/epi.12330

[19] O’Muircheartaigh J, Vollmar C, Barker GJ, et al Focal structural changes and cognitive dysfunction in juvenile myoclonic epilepsy. Neurology. 2011;76(1):34–40. 21205693 10.1212/WNL.0b013e318203e93d PMC3030222

[20] O’Muircheartaigh J, Vollmar C, Barker GJ, et al Abnormal thalamocortical structural and functional connectivity in juvenile myoclonic epilepsy. Brain. 2012;135(Pt 12):3635–3644. 23250883 10.1093/brain/aws296 PMC3525058

[21] Struck AF, Garcia-Ramos C, Prabhakaran V, et al Motor-associated thalamic nuclei are reduced in juvenile myoclonic epilepsy. Epilepsia. 2025;66:4381–4393. 40682576 10.1111/epi.18571 PMC12661284

[22] Tustison NJ, Avants BB, Cook PA, et al N4ITK: Improved N3 bias correction. IEEE Trans Med Imaging. 2010;29(6):1310–1320. 20378467 10.1109/TMI.2010.2046908 PMC3071855

[23] Iglesias JE, Insausti R, Lerma-Usabiaga G, et al A probabilistic atlas of the human thalamic nuclei combining ex vivo MRI and histology. Neuroimage. 2018;183:314-326. doi: 10.1016/j.neuroimage.2018.08.012 PMC6215335 30121337

[24] Tregidgo HFJ, Soskic S, Althonayan J, et al Accurate Bayesian segmentation of thalamic nuclei using diffusion MRI and an improved histological atlas. Neuroimage. 2023;274:120129. 37088323 10.1016/j.neuroimage.2023.120129 PMC10636587

[25] Dinga R, Fraza CJ, Bayer JMM, et al Normative modeling of neuroimaging data using generalized additive models of location scale and shape. *Biorxiv*. [Preprint]. 2021. doi: 10.1101/2021.06.14.448106

[26] Whelan CD, Altmann A, Botía JA, et al Structural brain abnormalities in the common epilepsies assessed in a worldwide ENIGMA study. Brain. 2018;141(2):391–408. 29365066 10.1093/brain/awx341 PMC5837616

[27] Sørensen Ø, Brandmaier AM, Macià D, et al Meta-analysis of generalized additive models in neuroimaging studies. NeuroImage. 2021;224:117416. 33017652 10.1016/j.neuroimage.2020.117416

[28] Bernhardt BC, Bernasconi N, Concha L, Bernasconi A. Cortical thickness analysis in temporal lobe epilepsy: Reproducibility and relation to outcome. Neurology. 2010;74(22):1776–1784. 20513813 10.1212/WNL.0b013e3181e0f80a

[29] Widjaja E, Mahmoodabadi SZ, Snead OC, et al Widespread cortical thinning in children with frontal lobe epilepsy. Epilepsia. 2011;52(9):1685–1691. 21627647 10.1111/j.1528-1167.2011.03085.x

[30] Benjamini YHW . Controlling the false discovery rate: A practical and powerful approach to multiple testing. Journal of the Royal Statistical Soceity. 1995;57(1):289–300.

[31] Hennig C . Cluster-wise assessment of cluster stability. Comput Stat Data Anal. 2007;52(1):258–271.

[32] Kind AJH, Jencks S, Brock J, et al Neighborhood socioeconomic disadvantage and 30-day rehospitalization: A retrospective cohort study. Ann Intern Med. 2014;161(11):765–774. 25437404 10.7326/M13-2946 PMC4251560

[33] Struck AF, Garcia-Ramos C, Prabhakaran V, et al Latent cognitive phenotypes in juvenile myoclonic epilepsy: Clinical, sociodemographic, and neuroimaging associations. Epilepsia. 2025;66(1):253–264. 39487825 10.1111/epi.18167 PMC11742545

[34] Devinsky O, Elder C, Sivathamboo S, et al Idiopathic generalized epilepsy: Misunderstandings, challenges, and opportunities. Neurology. 2024;102(3):e208076. 38165295 10.1212/WNL.0000000000208076 PMC11097769

[35] Usui N, Kotagal P, Matsumoto R, et al Focal semiologic and electroencephalographic features in patients with juvenile myoclonic epilepsy. Epilepsia. 2005;46(10):1668–1676. 16190941 10.1111/j.1528-1167.2005.00262.x

[36] Woermann FG, Free SL, Koepp MJ, et al Abnormal cerebral structure in juvenile myoclonic epilepsy demonstrated with voxel-based analysis of MRI. Brain. 1999;122(11):2101–2108. 10545395 10.1093/brain/122.11.2101

[37] Kim JH, Lee JK, Koh S-B, et al Regional grey matter abnormalities in juvenile myoclonic epilepsy: A voxel-based morphometry study. Neuroimage. 2007;37(4):1132–1137. 17689105 10.1016/j.neuroimage.2007.06.025

[38] Crespo Pimentel B, Kuchukhidze G, Xiao F, et al Sodium valproate is associated with cortical thinning of disease-specific areas in juvenile myoclonic epilepsy. J Neurol Neurosurg Psychiatry. 2024;96(1):11–14. 39043568 10.1136/jnnp-2024-333703

[39] de Nijs L, Wolkoff N, Coumans B, et al Mutations of EFHC1, linked to juvenile myoclonic epilepsy, disrupt radial and tangential migrations during brain development. Hum Mol Genet. 2012;21(23):5106–5117. 22926142 10.1093/hmg/dds356 PMC3490517

[40] Dazzo E, Santulli L, Posar A, et al Autosomal dominant lateral temporal epilepsy (ADLTE): Novel structural and single-nucleotide LGI1 mutations in families with predominant visual auras. Epilepsy Res. 2015;110:132–138. 25616465 10.1016/j.eplepsyres.2014.12.004

[41] Becchetti A, Aracri P, Meneghini S, et al The role of nicotinic acetylcholine receptors in autosomal dominant nocturnal frontal lobe epilepsy. Front Physiol. 2015;6:22. 25717303 10.3389/fphys.2015.00022 PMC4324070

[42] Berkovic SF, Izzillo P, McMahon JM, et al LGI1 mutations in temporal lobe epilepsies. Neurology. 2004;62(7):1115–1119. 15079010 10.1212/01.wnl.0000118213.94650.81

[43] Cardozo PL, de Lima IBQ, Maciel EMA, Silva NC, Dobransky T, Ribeiro FM. Synaptic elimination in neurological disorders. Curr Neuropharmacol. 2019;17(11):1071–1095. 31161981 10.2174/1570159X17666190603170511 PMC7052824

[44] Smith ML . Rethinking cognition and behavior in the new classification for childhood epilepsy: Examples from frontal lobe and temporal lobe epilepsies. Epilepsy Behav. 2016;64(Pt B):313–317. 27346387 10.1016/j.yebeh.2016.04.050

[45] Ratcliffe C, Wandschneider B, Baxendale S, et al Cognitive function in genetic generalized epilepsies: Insights from neuropsychology and neuroimaging [internet]. Front Neurol. 2020;11:144. [cited 2020 Mar 19] Available from: https://www.frontiersin.org/articles/10.3389/fneur.2020.00144/full 32210904 10.3389/fneur.2020.00144 PMC7076110

[46] Loughman A, Bowden SC, D’Souza WJ. A comprehensive assessment of cognitive function in the common genetic generalized epilepsy syndromes. Eur J Neurol. 2017;24(3):453–460. 28026919 10.1111/ene.13232

[47] Garcia-Ramos C, Dabbs K, Lin JJ, et al Progressive dissociation of cortical and subcortical network development in children with new-onset juvenile myoclonic epilepsy. Epilepsia. 2018;59(11):2086–2095. 30281148 10.1111/epi.14560 PMC6334640

[48] Kim JH . Grey and white matter alterations in juvenile myoclonic epilepsy: A comprehensive review. J Epilepsy Res. 2017;7(2):77–88. 29344465 10.14581/jer.17013 PMC5767493

[49] Syvertsen M, Selmer K, Enger U, et al Psychosocial complications in juvenile myoclonic epilepsy. Epilepsy Behav. 2019;90:122–128. 30530133 10.1016/j.yebeh.2018.11.022

[50] Blumenfeld H, Varghese GI, Purcaro MJ, et al Cortical and subcortical networks in human secondarily generalized tonic–clonic seizures. Brain. 2009;132(4):999–1012. 19339252 10.1093/brain/awp028 PMC2724910

[51] Paus T, Keshavan M, Giedd JN. Why do many psychiatric disorders emerge during adolescence? Nat Rev Neurosci. 2008;9(12):947–957. 19002191 10.1038/nrn2513 PMC2762785

[52] Polack P-O, Guillemain I, Hu E, et al Deep layer somatosensory cortical neurons initiate spike-and-wave discharges in a genetic model of absence seizures. J Neurosci. 2007;27(24):6590–6599. 17567820 10.1523/JNEUROSCI.0753-07.2007 PMC6672429

