# Supplementary material for: Juvenile myoclonic epilepsy heterogeneity uncovered: Z-mapped imaging endophenotypes of cortical and subcortical structures and their clinical, cognitive and psychiatric features
Source: Brain Commun. 2026 Mar 24;8(2):fcag107. doi: 10.1093/braincomms/fcag107 (PMC13042235; doi:10.1093/braincomms/fcag107)
Supplement: fcag107_Supplementary_Data [file fcag107_supplementary_data.docx]

**Supplementary Section**

**Supplementary Section 1: Grouping of Desikan-Killiany Parcellation and FreeSurfer Segmentation Regions**. This section details the grouping of regions from the Desikan-Killiany (DK) cortical parcellation and FreeSurfer subcortical segmentation into larger, composite regions for analysis. The groupings are based on anatomical and functional considerations, combining individual regions from the DK parcellation and FreeSurfer segmentation into broader categories.

**Thalamic Groups**

- **Left Motor Thalamus**:
  - Ventral Anterior Nucleus (Left)
  - Ventral Lateral Anterior Nucleus (Left)
  - Ventral Lateral Posterior Nucleus (Left)
- **Right Motor Thalamus**:
  - Ventral Anterior Nucleus (Right)
  - Ventral Lateral Anterior Nucleus (Right)
  - Ventral Lateral Posterior Nucleus (Right)
- **Left Non-Motor Thalamus**:
  - Lateral Geniculate Nucleus (Left)
  - Medial Geniculate Nucleus (Left)
  - Pulvinar Nucleus (Left)
  - Medial Pulvinar Nucleus (Left)
  - Lateral Superior Geniculate Nucleus (Left)
  - Ventral Posterior Lateral Nucleus (Left)
  - Centromedian Nucleus (Left)
  - Anterior Pulvinar Nucleus (Left)
  - Mediodorsal Medial Nucleus (Left)
  - Parafascicular Nucleus (Left)
  - Ventral Anterior Magnocellular Nucleus (Left)
  - Mediodorsal Intermediate Nucleus (Left)
  - Central Medial Nucleus (Left)
  - Medial Ventral Reuniens Nucleus (Left)
  - Ventral Medial Nucleus (Left)
  - Central Lateral Nucleus (Left)
  - Second Pulvinar Nucleus (Left)
  - Posterior Thalamic Nucleus (Left)
  - Anterior Ventral Nucleus (Left)
  - Posterior Commissural Nucleus (Left)
  - Lateral Posterior Nucleus (Left)
  - Lateral Dorsal Nucleus (Left)
- **Right Non-Motor Thalamus**:
  - Lateral Geniculate Nucleus (Right)
  - Medial Geniculate Nucleus (Right)
  - Pulvinar Nucleus (Right)
  - Medial Pulvinar Nucleus (Right)
  - Lateral Superior Geniculate Nucleus (Right)
  - Ventral Posterior Lateral Nucleus (Right)
  - Centromedian Nucleus (Right)
  - Anterior Pulvinar Nucleus (Right)
  - Mediodorsal Medial Nucleus (Right)
  - Parafascicular Nucleus (Right)
  - Ventral Anterior Magnocellular Nucleus (Right)
  - Mediodorsal Intermediate Nucleus (Right)
  - Central Medial Nucleus (Right)
  - Medial Ventral Reuniens Nucleus (Right)
  - Ventral Medial Nucleus (Right)
  - Central Lateral Nucleus (Right)
  - Second Pulvinar Nucleus (Right)
  - Posterior Thalamic Nucleus (Right)
  - Anterior Ventral Nucleus (Right)
  - Posterior Commissural Nucleus (Right)
  - Lateral Posterior Nucleus (Right)
  - Lateral Dorsal Nucleus (Right)

**Subcortical Groups**

- **Left Subcortical Grey**:
  - Caudate (Left)
  - Putamen (Left)
  - Pallidum (Left)
  - Accumbens Area (Left)
  - Thalamus (Left)
  - Hippocampus (Left)
  - Amygdala (Left)
  - Cerebellum Cortex (Left)
- **Right Subcortical Grey**:
  - Caudate (Right)
  - Putamen (Right)
  - Pallidum (Right)
  - Accumbens Area (Right)
  - Thalamus (Right)
  - Hippocampus (Right)
  - Amygdala (Right)
  - Cerebellum Cortex (Right)
- **Left Subcortical White**:
  - Cerebellum White Matter (Left)
- **Right Subcortical White**:
  - Cerebellum White Matter (Right)

**Cortical Groups**

- **Left Motor** (DK Parcellation):
  - Precentral Gyrus
  - Superior Frontal Gyrus
  - Caudal Middle Frontal Gyrus
  - Pars Opercularis
- **Right Motor** (DK Parcellation):
  - Precentral Gyrus
  - Superior Frontal Gyrus
  - Caudal Middle Frontal Gyrus
  - Pars Opercularis
- **Left Frontal Non-Motor** (DK Parcellation):
  - Lateral Orbitofrontal Cortex
  - Medial Orbitofrontal Cortex
  - Pars Orbitalis
  - Pars Triangularis
  - Rostral Middle Frontal Gyrus
  - Frontal Pole
- **Right Frontal Non-Motor** (DK Parcellation):
  - Lateral Orbitofrontal Cortex
  - Medial Orbitofrontal Cortex
  - Pars Orbitalis
  - Pars Triangularis
  - Rostral Middle Frontal Gyrus
  - Frontal Pole
- **Left Parietal** (DK Parcellation):
  - Inferior Parietal Cortex
  - Paracentral Lobule
  - Postcentral Gyrus
  - Precuneus
  - Superior Parietal Cortex
  - Supramarginal Gyrus
- **Right Parietal** (DK Parcellation):
  - Inferior Parietal Cortex
  - Paracentral Lobule
  - Postcentral Gyrus
  - Precuneus
  - Superior Parietal Cortex
  - Supramarginal Gyrus
- **Left Temporal** (DK Parcellation):
  - Banks of the Superior Temporal Sulcus
  - Entorhinal Cortex
  - Fusiform Gyrus
  - Inferior Temporal Gyrus
  - Middle Temporal Gyrus
  - Parahippocampal Gyrus
  - Superior Temporal Gyrus
  - Temporal Pole
  - Transverse Temporal Gyrus
- **Right Temporal** (DK Parcellation):
  - Banks of the Superior Temporal Sulcus
  - Entorhinal Cortex
  - Fusiform Gyrus
  - Inferior Temporal Gyrus
  - Middle Temporal Gyrus
  - Parahippocampal Gyrus
  - Superior Temporal Gyrus
  - Temporal Pole
  - Transverse Temporal Gyrus
- **Left Occipital** (DK Parcellation):
  - Cuneus
  - Lateral Occipital Cortex
  - Lingual Gyrus
  - Pericalcarine Cortex
- **Right Occipital** (DK Parcellation):
  - Cuneus
  - Lateral Occipital Cortex
  - Lingual Gyrus
  - Pericalcarine Cortex
- **Left Limbic** (DK Parcellation):
  - Caudal Anterior Cingulate Cortex
  - Isthmus Cingulate Cortex
  - Posterior Cingulate Cortex
  - Rostral Anterior Cingulate Cortex
  - Insula
- **Right Limbic** (DK Parcellation):
  - Caudal Anterior Cingulate Cortex
  - Isthmus Cingulate Cortex
  - Posterior Cingulate Cortex
  - Rostral Anterior Cingulate Cortex
  - Insula

**Supplementary Section 2A: Subcortical mean Z-score and T-statistics (JME N=62 vs controls N=41).**

(**A**) Mean subcortical Z-scores in the JME group. (**B**) T-statistics from two-sample t-tests comparing JME vs controls for each region. ****** indicates FDR-adjusted p<0.05 across subcortical regions.


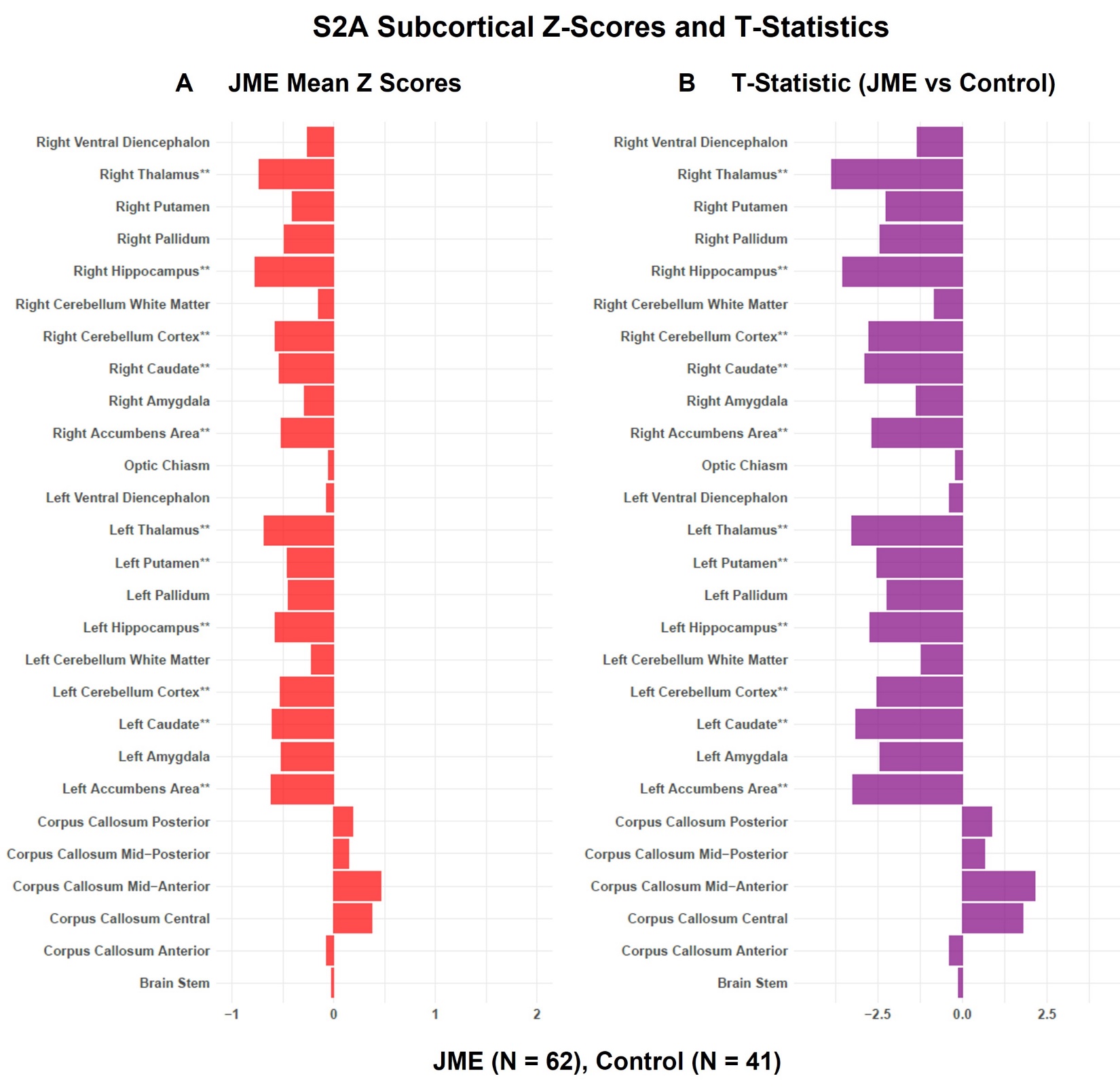


**Supplementary Section 2B: Thalamic Nuclei Z-scores and T-Statistics (JME N=62 vs controls N=41).**

(**A**) Mean thalamic nuclei Z-scores in the JME group. (**B**) T-statistics from two-sample t-tests comparing JME vs controls for each nucleus. ****** indicates FDR-adjusted p<0.05 across thalamic nuclei.


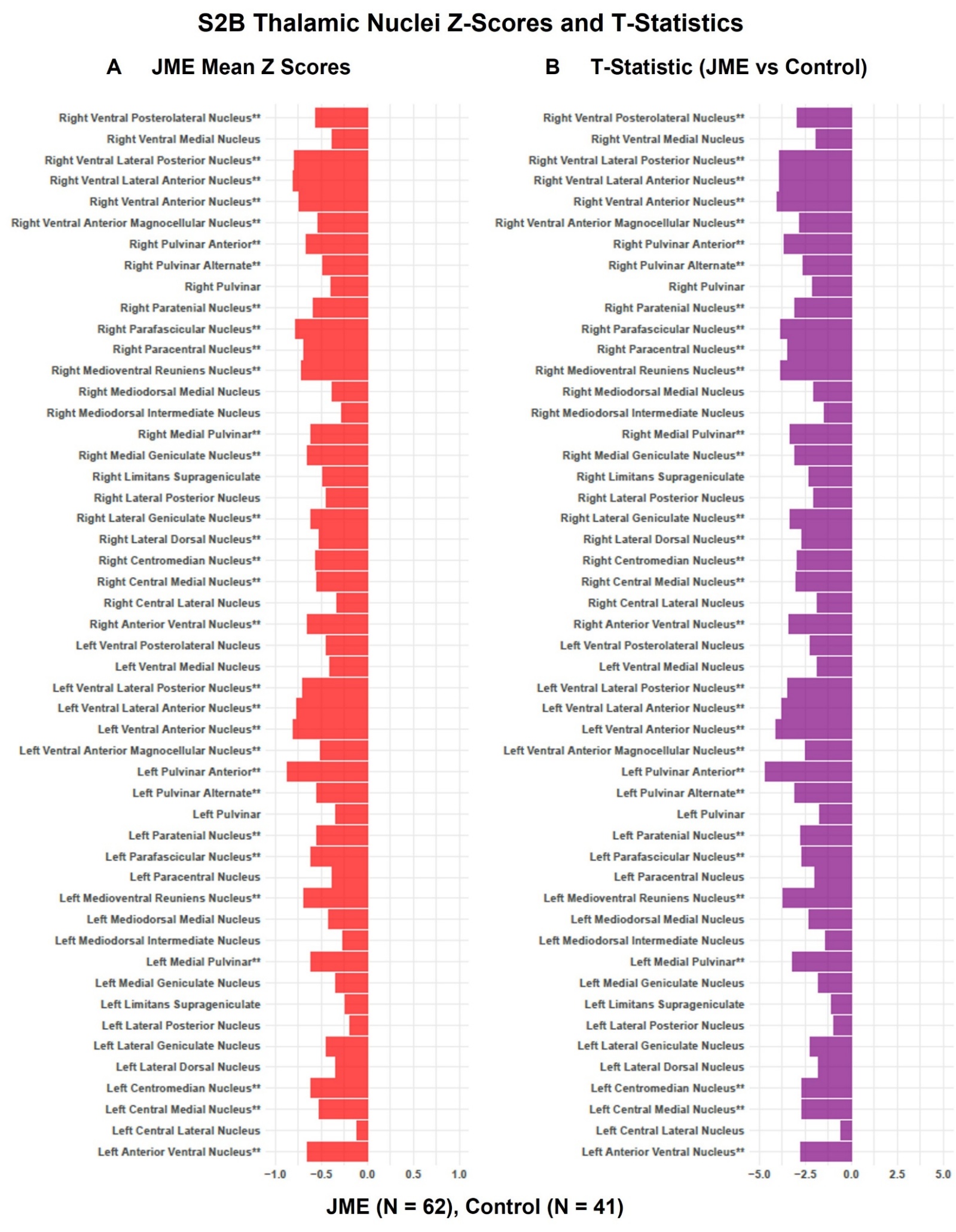


**Supplementary Section 3:** Comparison of Cluster Stability between of the mean Jaccard Coefficient with 500 bootstrapped trials for K-means and Hierarchical Clustering for range of clusters between 2 and 5.

**
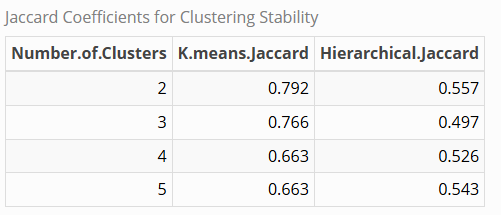
**

**Supplementary Figure 1A: Elbow method for selecting k in k-means clustering.** K-means clustering was applied to regional Z-score features from JME participants (N=62). The plot shows total within-cluster sum of squares as a function of the number of clusters (k); the elbow/inflection suggests diminishing returns beyond k=3.


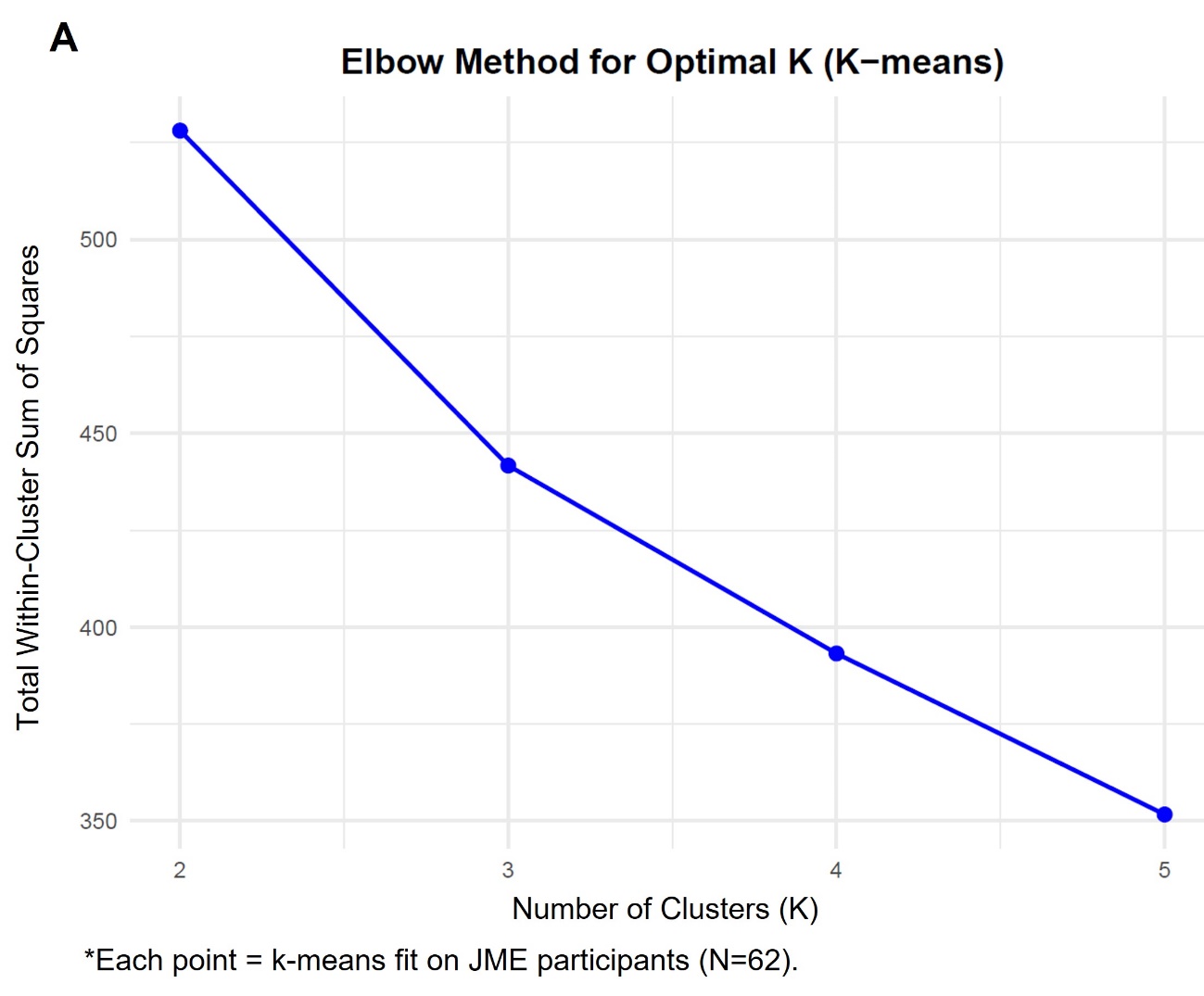


**Supplementary Figure 1B: Gap statistic for selecting k in k-means clustering.** Gap statistic was computed for k-means clustering of regional Z-score features from JME participants (N = 62). Points show the gap statistic for each k; error bars indicate ±1 standard error (SE). The criterion supports k = 3 as the preferred solution.

**
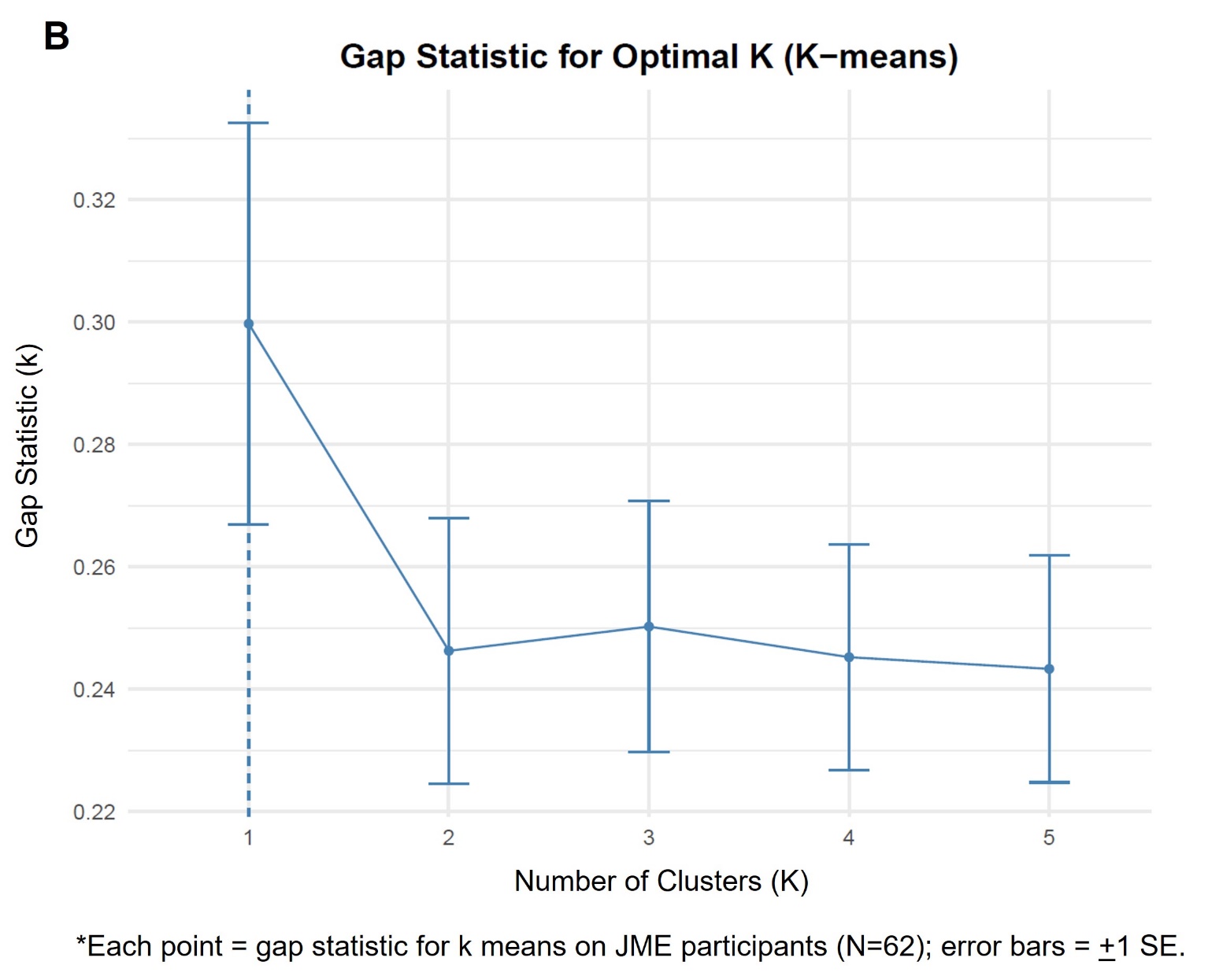
**

**Supplementary Figure 2. Regional Z-Scores across imaging endophenotypes and controls.** Boxplots with overlaid participant-level data showing regional Z-scores for controls (N=41) and JME participants (N=62) stratified by endophenotype. Each JME cluster was compared to the healthy controls using uncorrected p-values from Wilcoxon Test. NS: not significant, ***<0.001, **<0.01, *<0.05. Premotor is equivalent to motor used in other sections of the manuscript incorporating primary motor cortex and pre-motor frontal regions. Light blue is the control (N=41), blue green is the Subcortical Reduction group (N=27), Orange is the Increase Cortical Thickness group (N=21), and purple is the Decreased Cortical Thickness group (N=14).

**
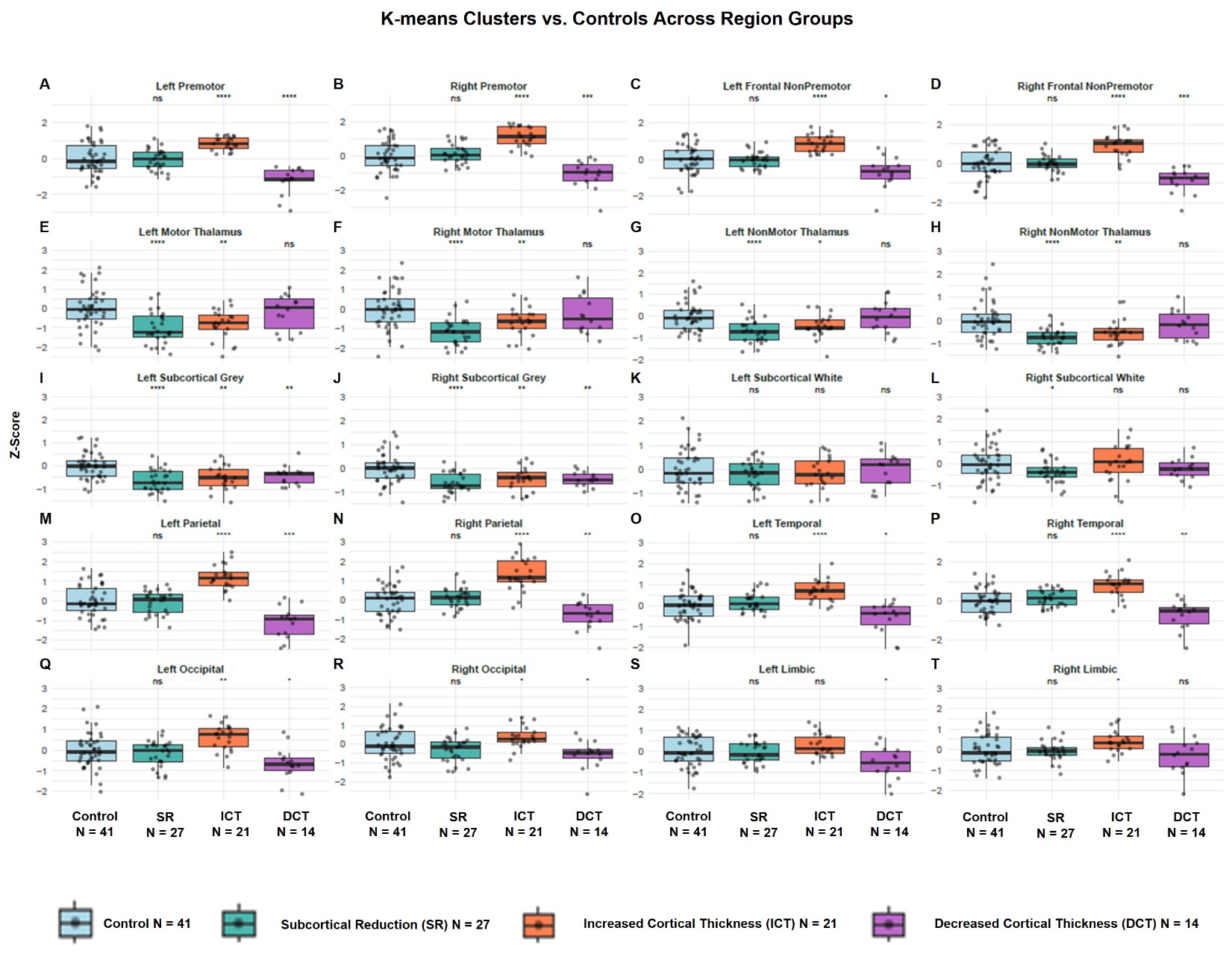
**
